# JAK2 gene knockout inhibits corneal allograft rejection in mice by regulating dendritic cell-induced T cell immune tolerance

**DOI:** 10.1038/s41420-022-01067-5

**Published:** 2022-06-16

**Authors:** Jun Hu, Wanping Zhang, Lingjuan Xu, Lihua Hu

**Affiliations:** 1 Aier Eye Hospital of Wuhan University, Wuhan, 430063 P. R. China; 2 Ophthalmology Department, Tongji Hospital, Tongji Medical College, Huazhong University of Science and Technology, Wuhan, 430030 P. R. China

**Keywords:** Cell biology, Corneal diseases

## Abstract

Corneal allograft rejection can be seen in some patients after corneal transplantation. The present study intends to investigate whether JAK2 gene knockout affects corneal allograft rejection through regulation of dendritic cells (DCs)-induced T cell immune tolerance. In order to identify the target gene related to corneal allograft rejection, high-throughput mRNA sequencing and bioinformatics analysis were performed. JAK2 knockout mice were constructed and subjected to corneal allograft transplantation. The incidence of immune rejection was observed, the percentage of CD4^+^ T cells was detected, and the expression of Th1 cytokine interferon γ (IFN-γ) was determined. Flow cytometry and ELISA were performed to analyze the effects of JAK2 gene knockout on bone marrow-derived DCs (BMDCs). JAK2 was the target gene related to corneal allograft rejection. JAK2 gene knockout contributed to significantly prolonged survival time of corneal grafts in mice and inhibited corneal allograft rejection. The in vitro cell experiment further confirmed that JAK2 gene knockout contributed to the inactivation of CD4^+^ T cells and induced IFN-γ expression, accompanied by inhibition of DC immune function, development, maturation, and secretion of inflammatory cytokines. Collectively, JAK2 gene knockout inactivates CD4^+^ T cells to decrease IFN-γ expression, as well as inhibits DC development, maturation, and secretion of inflammatory cytokines, thereby reducing corneal allograft rejection.

## Introduction

Corneal transplantation is regarded as a prevalent solid organ transplantation that may encounter a failure due to T cell-mediated rejection [1]. Patients with corneal neovascularization or edema, and large donor graft buttons may be more vulnerable to treatment failure due to corneal allograft rejection [2]. Currently, the widely used immunosuppressive agents for preventing corneal graft rejection mainly include steroids [3]. Importantly, it has been reported that immunomodulatory therapies targeting dendritic cells (DCs), an important player of the immune system, can improve the survival of corneal grafts [4]. Strikingly, the potential of gene therapy has been highlighted in cornea transplantation through modification of allografts ex vivo before transplantation [5]. Against such a backdrop, it is of significance to search target genes for control of corneal allograft rejection.

Janus kinase 2 (JAK2) is identified as a cytoplasmic tyrosine kinase that plays an important role in cytokine signaling [6]. Intriguingly, it is known that the increase of JAK expression is related to the immune rejection of allografts as well as the inflammation in autoimmune diseases [7]. As previously reported, JAK2, as a key modulator of the immune response, is involved in the occurrence of graft-versus-host disease, a contributor to transplant-related mortality following allogeneic hematopoietic cell transplantation [8]. Activated JAK2 by treatment of cryptotanshinone could regulate CD4^+^ T cell cytotoxicity in lung tumor [9]. Of note, it was found that JAK2 could modulate DC differentiation and that the inhibition of JAK2 could suppress inflammatory dendritic epidermal cell development and function in atopic dermatitis [10]. The abnormal activation of JAK2 signaling in immature myeloid DCs was shown to participate in the regulation of immune tolerance [11]. Besides, it was revealed that knockout of JAK2 selectively inhibited DCs-mediated innate immunity in a mouse model of lipopolysaccharide (LPS)-induced septic shock [12]. Inhibition of JAK2 could bring about long-term tolerance to alloantigen by DCs-induced T cells [13]. Interferon γ (IFN-γ) production plays a crucial part in the process of corneal allograft rejection [14], and activated JAK2 could induce the expression of IFN-γ in tuberculosis [15]. Considering all the above findings, we proposed the hypothesis in this study that JAK2 gene knockout could affect corneal allograft rejection, which involved with the regulation of DCs-induced T cell immune tolerance.

## Results

### Twenty-four target genes related to corneal allograft rejection were screened through high-throughput sequencing

**Fig. 1:**
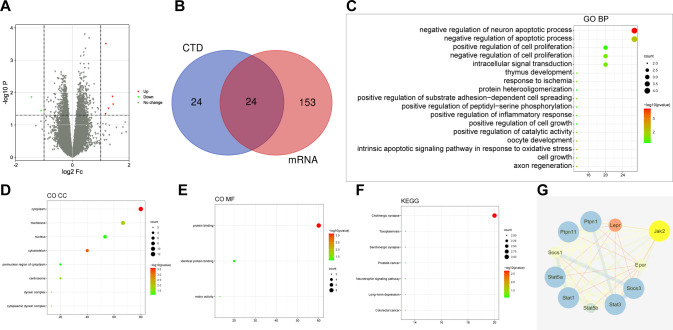
Twenty-four target genes related to corneal allograft rejection are screened through transcriptome high-throughput sequencing. **A** Differential expression of mRNAs in corneal allograft rejection and normal samples. The green dots indicate downregulation, the red dots indicate upregulation, and the gray dots indicate no significant difference. The *X*-axis represents the logarithm (log_2_) of the FoldChange between different groups, i.e., log_2_ (FC). The *Y*-axis represents the logarithm (−log_10_) of the *p* value, namely −log_10_ (*p* value). **B** The Venn diagram for the retrieval results of the two databases. **C** GO functional analysis of DEmRNA at the biological process level. **D** The GO function of DEmRNA was analyzed at the cellular component level. **E** GO functional analysis of DEmRNA at the molecular function level. **F** The size of dots indicates the number of selected genes, and the color represents the *p* value of enrichment analysis. **G** Network for candidate target gene interaction (node represents protein, edge represents protein association, and colors and shapes represent degree value and Combine Score value.

In order to study the target genes related to the occurrence of corneal allograft rejection, we first used a high-throughput sequencing method for transcriptome analysis on normal control mice and model mice receiving corneal allografts. The results screened out a total of 177 DEmRNAs (90 upregulated DEmRNAs and 87 downregulated DEmRNAs) (Fig. 1A and Table S1). At the same time, 24 differential genes were obtained by intersection with the DEmRNAs with 48 genes related to corneal allograft rejection found in the CTD database (Fig. 1B).

Furthermore, GO and KEGG analyses were performed on 24 differentially expressed genes. The results of GO functional analysis showed that the target genes were mainly enriched in the items of negative regulation of the neural apoptotic process, negative regulation of the apoptotic process, oocyte development, and so on (Fig. 1C). In the cell component, they were mainly enriched in the cytoplasm, cytoskeleton, membrane, and other items (Fig. 1D). In molecular function, they were mainly enriched in protein binding, motor activity, and identical protein binding (Fig. 1E). KEGG pathway analysis revealed that the target genes were mainly enriched in items such as cholinergic synapse, long-term depression, colorectal cancer, prostate cancer, toxoplasmosis, and neurotrophin signaling pathway (Fig. 1F). These results suggest that candidate target genes mainly play a role in biological processes such as apoptosis and development, and are enriched in structures such as cytoplasm and cytoskeleton. The molecular function of candidate target genes is mainly involved in protein binding.

A total of 24 candidate target genes were introduced into the String database (species: mice) to obtain the protein-protein interaction (PPI) relationship. The PPI network was constructed using the Cytoscape software, involving 15 nodes and 15 edges (PPI enrichment *p* value < 1.0e^−16^). Subsequently, MCC network topology algorithm in the cytoHubba software was used to predict the top 10 hub genes from the PPI network, in which JAK2 was found to be the top 1 hub gene (Fig. 1G). Overall, JAK2 plays an important role in the regulation of corneal allograft rejection, so we chose JAK2 as the target gene for further study.

### JAK2 gene knockout significantly prolonged the survival time of corneal grafts

**Fig. 2:**
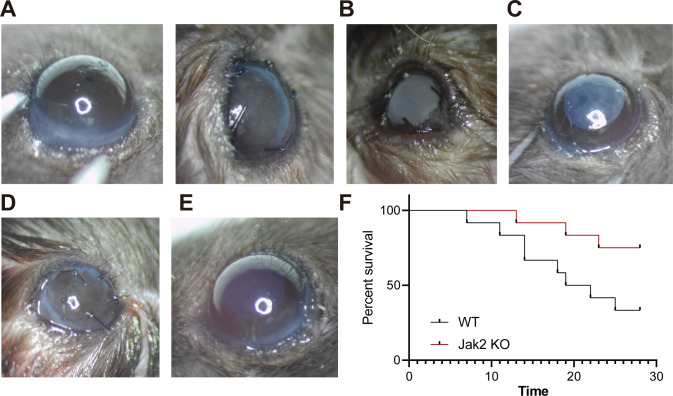
JAK2 gene knockout significantly prolongs the survival time of corneal grafts. **A** The corneas of mice before and after the operation (The left side is the cornea of mice before the operation. The right side is the cornea of mice after the operation. **B** Corneal rejection that occurred in WT mice 7 days after the operation. **C** Corneal rejection that occurred in WT mice 14 days after the operation. **D** Corneas without rejection in JAK2 knockout mice 7 days after the operation. **E** Corneas without rejection in JAK2 knockout mice 14 days after the operation. **F** The survival curve of corneal grafts 28 days after operation in WT and JAK2 knockout mice.

In order to verify the important role of JAK2 in early corneal allograft rejection, we successfully established the corneal allograft model in JAK2 knockout mice and WT mice, and compared the eyeballs of the mice before operation with those of the mice after modeling (Fig. 2A). Postoperative observation under a slit lamp showed that in WT mice, transient edema and opacity occurred due to inflammatory reaction 28 days after operation; two cases of corneal grafts were turbid 7 days after the operation, and the pupil was not visible (Fig. 2B). After removal of the corneal suture, the edema and opacity aggravated; 14 days after the operation, four cases of corneas showed edema, opacity, and completely opaque (Fig. 2C). Up to 21 days after the operation, 10 cases had corneal edema and opacity, and the pupil was not visible. The average survival time was (20.16) days; four cases of the cornea had long-term survival. In JAK2 knockout mice, transient corneal edema and opacity occurred due to inflammatory reaction. The corneal grafts were transparent 7 days after the operation (Fig. 2D). Only two cases had mild corneal epithelial edema. After the removal of the corneal suture, the cornea became transparent gradually. All corneal grafts were still transparent 14 days after the operation (Fig. 2E). Up to 21 days after the operation, only four cases had corneal opacity. The average survival time of the JAK2 knockout mice was (25.58) days; nine cases of corneal long-term survival. There was a significant difference in median survival time between WT mice and JAK2 knockout mice (*p* < 0.05).

WT mice and JAK2 knockout mice received donor corneas from BALB/c mice. The survival time of corneal grafts in JAK2 knockout mice was significantly prolonged (*p* = 0.0377). Forty days after the operation, the survival rate of corneal grafts in JAK2 knockout mice was 75.00%, while that in WT mice was only 33.33% (Fig. 2F).

These results suggest that JAK2 gene knockout can significantly prolong the survival time of corneal grafts, and JAK2 may be a key gene involved in the occurrence of corneal allograft rejection.

### JAK2 gene knockout prevented local corneal allograft rejection

**Fig. 3:**
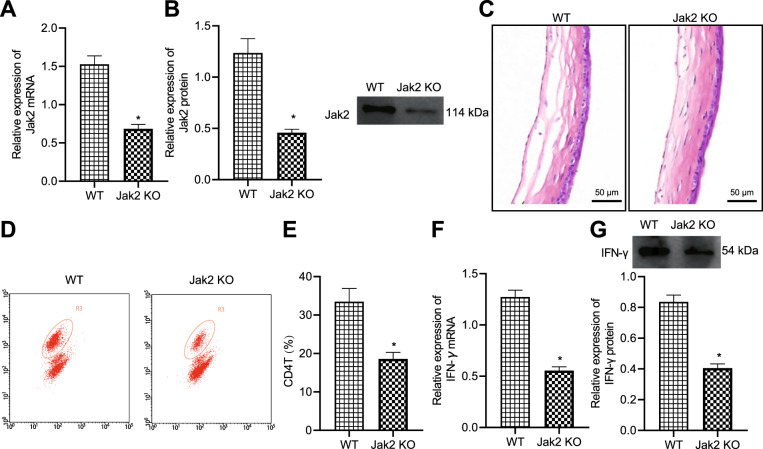
JAK2 gene knockout prevents local corneal allograft rejection. **A** The mRNA expression of JAK2 in corneal grafts 14 days after the operation. **B** The protein expression of JAK2 in corneal allografts 14 days after the operation. **C** HE staining for mouse corneas 14 days after the operation, scale bar = 50 μm. **D** The scatter plot for a percentage of CD4^+^ T cells in ipsilateral cervical lymph nodes of WT and JAK2 knockout mice 14 days after the operation. **E** The percentage of CD4^+^ T cells in ipsilateral cervical lymph nodes of WT mice and JAK2 knockout mice 14 days after the operation. **F** The mRNA expression of IFN-γ in corneal grafts of WT mice and JAK2 knockout mice 14 days after the operation. **G** The mRNA expression of IFN-γ in corneal grafts of WT mice and JAK2 knockout mice 14 days after the operation. *p < 0.05 vs. compared with WT mice.

Immune rejection after corneal transplantation is the critical reason for the failure of corneal grafts to survive [16]. After model establishment, the differences in terms of the corneal graft and inflammatory infiltrating cells, CD4^+^ T cells, and IFN-γ expression were compared, in order to investigate the relationship between JAK2 and local corneal allograft rejection. RT-qPCR and Western blot revealed that the mRNA and protein expression of JAK2 in the corneal allografts of WT mice was significantly higher than that in JAK2 knockout mice (Fig. 3A, B).

Based on the results of HE staining for corneal allografts 14 days after the operation, the WT mice revealed rejected corneal graft with edema, thickening, a structural disorder of stromal layer (normally composed of clear-cut epithelial cells and regular arrangement of collagen fibers), neovascular lumen and a large number of inflammatory cell infiltration. Corneal allografts in JAK2 knockout mice showed mild edema, along with only a little inflammatory cell infiltration (Fig. 3C).

The detection of the percentage of CD4^+^ T cells in the ipsilateral cervical lymph nodes of the operated mouse eyes showed that compared with JAK2 knockout mice, WT mice had a notable increase in the number of CD4^+^ T cells in the ipsilateral cervical lymph nodes of the operated eyes (Fig. 3D, E). In addition, determination of IFN-γ expression in corneal allografts detected by RT-qPCR and Western blot 14 days after operation suggested that the expression of IFN-γ in JAK2 knockout mice was significantly higher than that in JAK2 knockout mice (Fig. 3F, G).

Taken together, the occurrence of local corneal allograft rejection can be prevented through JAK2 knockout.

### JAK2 gene knockout inhibited the development, maturation, and secretion of inflammatory cytokines of DCs

**Fig. 4:**
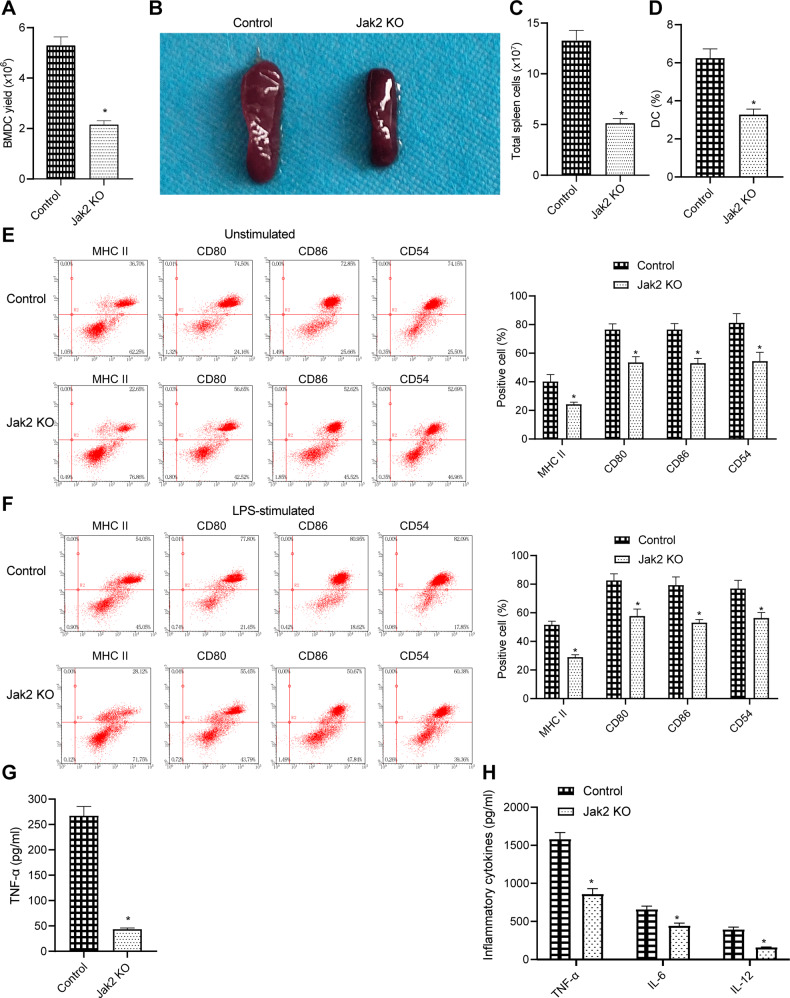
JAK2 gene knockout inhibits the development, maturation, and secretion of inflammatory cytokines of DCs. **A** After two weeks of induction of gene knockout, BMDCs were induced by GM-CSF and IL-4. BMDCs were collected and counted on the 10th day of culture. **B** Two weeks after JAK2 gene knockout, mouse spleens were isolated and weighed. **C** Spleens were isolated from JAK2 knockout mice and normal control mice, single-cell suspension was prepared, and the total number of spleen cells was counted after red blood cells were lysed with 1 × ACK red blood cell lysate. **D** The cell suspension of spleens from JAK2 knockout mice and normal control mice. CD11c staining was performed, followed by flow cytometry. **E** BMDCs were collected after 2 weeks of induction of gene knockout. GM-CSF and IL-4 were used to induce BMDCs, which were collected at the 10th day of culture. After staining with CD11c, MHC-II, and costimulatory molecules (CD80, CD86, and CD54) antibodies, flow cytometry was performed. The left side shows the representative flow chart, and the right side is the statistical chart. **F** BMDCs were collected after 2 weeks of induction of gene knockout. BMDCs were stimulated with LPS for 24 h on the 9th day of culture and then collected for flow cytometry. The left side is the representative flow chart, and the right side is the statistical chart. **G** BMDCs from JAK2 knockout mice and normal control mice were seeded into 96 well plates at the same density on the 9^th^ day of culture. After 24 h, the culture supernatant was collected. ELISA was performed to determine the expression of TNF-α secreted by BMDCs from JAK2 knockout mice. **H** BMDCs from JAK2 knockout mice and normal control mice were seeded into 96 well plates at the same density on the 9th day of culture, followed by LPS stimulation for 24 h. The culture supernatant was collected, and ELISA was performed to determine the expression of TNF-α, IL-6, and IL-12 secreted by BMDCs from JAK2 knockout mice. *p < 0.05 *vs*. compared with normal control mice.

We isolated BMDCs from the bone marrow of JAK2 knockout mice and normal control mice, and treated BMDCs with GM-CSF and IL-4. We found that JAK2 gene knockout markedly reduced the number of DCs than that in normal control mice (Fig. 4A).

In addition, we further detected the number of DCs in spleen tissue of JAK2 gene knockout mice and normal control mice and found that the spleen weight of JAK2 knockout mice was markedly lighter than that of normal control mice (Fig. 4B). We also found that the total cell number in spleen tissue in JAK2 knockout mice was notably less than that in normal control mice (Fig. 4C). On the premise of the decreased total cell number in spleen tissue, the proportion of DCs in spleen tissue of JAK2 knockout mice was also notably lower than that of normal control mice (Fig. 4D). These results suggest that JAK2 gene knockout is able to significantly inhibit the development of DCs.

Similarly, after treatment with GM-CSF and IL-4 for 9 days, part of the DCs was taken and stimulated with 500 ng/mL LPS for 24 h. On the 10th day, the cells were collected to detect the surface markers. Flow cytometry showed that the purity of DCs was more than 85% in both JAK2 knockout mice and normal control mice. By analyzing the phenotype of CD11C^+^ cells, we found that JAK2 gene knockout significantly reduced the response of DCs to induction of maturation. In the absence of stimulation, the expression of MHC class II molecules and costimulatory molecules (such as CD80, CD86, and CD54) on the surface of BMDCs from JAK2 knockout mice was significantly lower than that on the surface of controls (Fig. 4E). Under LPS stimulation, most of BMDCs from normal control mice entered mature state, showing high expression of MHC class II molecules and costimulatory molecules (CD80, CD86, and CD54). On the contrary, only a small number of BMDCs from JAK2 gene knockout entered mature state under LPS stimulation (Fig. 4F). To conclude, JAK2 gene knockout can significantly inhibit the maturation of BMDCs.

One of the important functions of DCs is to secrete cytokines to assist other cell differentiation and function [17]. According to the results from ELISA, before stimulation, IL-2, IL-6, IL-10, and IL-12 could not be detected but a low level of TNF-α in BMDC supernatant of JAK2 knockout mice and normal control mice. TNF-α secreted by BMDCs from JAK2 knockout mice was only about 1/10 of that from the WT mice (Fig. 4G). After LPS stimulation, BMDCs secreted a lot of inflammatory cytokines such as TNF-α, IL-6, and IL-12. Moreover, TNF-α, IL-6, and IL-12 secreted by BMDCs from JAK2 knockout mice were significantly lower than those from normal control mice (Fig. 4H). Collectively, JAK2 deficiency affects the secretion of inflammatory cell molecules by DCs.

In conclusion, JAK2 knockout is capable of inhibiting the innate immune function of DCs such as maturation and secretion of inflammatory cytokines.

## Discussion

Immunologic graft rejection is considered to be the major contributor to graft failure in corneal transplantation [3]. In the present study, we set out to explore the role of JAK2 gene knockout in the regulation of corneal allograft rejection, the results of which found that JAK2 gene knockout could suppress corneal allograft rejection by affecting DCs-induced T cell immune tolerance.

First of all, our transcriptome high-throughput sequencing screened out 24 target genes related to corneal allograft rejection, among which JAK2 was found to be the top 1 key gene in the PPI network. Moreover, our study further demonstrated that JAK2 gene knockout could significantly prolong the survival time of corneal grafts while also preventing the occurrence of local corneal allograft rejection. JAK2 was revealed to be implicated in the secretion of the chemokine IL-8 in human corneal fibroblasts [18]. JAK pathway is believed to be able to regulate myeloid-derived suppressor cells, which can affect immune tolerance, graft survival, and rejection [19]. It was reported that the use of AG490, an inhibitor of JAKs including JAK2, could regulate CD4^+^CD25^+^ T cell development as well as a Th2 shift of CD4^+^ T cells, thereby exerting prevention in acute lung allograft rejection in a rat model [20]. Of note, a previous study demonstrated that JAK2 was a potential biologic target for control of allograft rejection, for suppression of JAK2 could result in tolerance to alloantigen by human DCs-triggered T cells, involved with the regulation of memory T cells and responder Th1 and Th17 cells [13]. Besides, targeting JAK2 could decrease graft-versus-host disease as well as xenograft rejection by mediating T cell differentiation and is of potential to modulate donor alloreactivity following allogeneic hematopoietic cell transplantation or solid organ transplantation [8]. Pharmacologic suppression of JAK2 could diminish graft-versus-host disease and retain the graft-versus-leukemia effect in allogeneic hematopoietic stem cell transplantation, and was thus suggested to be applied in diseases including organ transplant rejection [21]. In addition, the recipient and donor JAK2 46/1 haplotypes were unfolded to be accountable for acute graft-versus-host disease after allogeneic hematopoietic stem cell transplantation [22]. The above findings can support our result regarding the regulation of corneal allograft rejection by JAK2 gene knockout.

Furthermore, the current study revealed that JAK2 gene knockout could inhibit DC development, maturation, and secretion of inflammatory cytokines. To our acknowledge, an increasing number of studies have unfolded the regulatory role of JAK2 in CD4^+^ T cells and DCs. JAK2-STAT3 activation was found in splenic CD4^+^ T cells and could affect cell differentiation, thereby aiding in modulating the inflammation [23]. It was unveiled that the lack of JAK2 selectively repressed DCs-regulated innate immunity in mice with LPS-induced septic shock by playing an important role in the development and maturation of DCs while also regulating DC secretion of proinflammatory cytokines [12]. In addition, the activation of the JAK2 signaling pathway by leptin could accelerate the migration and maturation of DCs in a mouse model [24]. Interestingly, the regulatory function of JAK2 on IFN-γ has been previously reported. For instance, silencing of JAK2 was unfolded to result in suppression of IFN-γ-induced activation of peripheral blood mononuclear cells from patients with STAT4 risk [25], and downregulation of JAK2 by miR-21 could repress IFN-γ-induced STAT1 pathway in macrophages [26]. Therefore, the function of JAK2 gene knockout in corneal allograft rejection was achieved by affecting DCs-induced T cell immune tolerance.

**Fig. 5:**
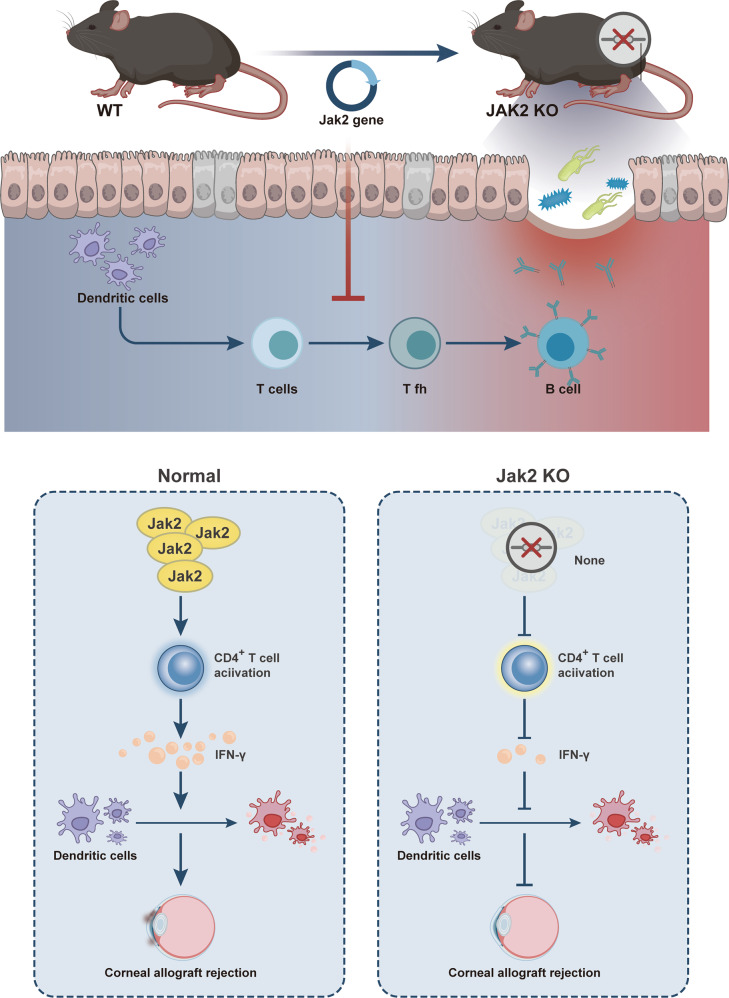
The molecular mechanism plot for the role of JAK2 gene knockout in regulating corneal allograft rejection. JAK2 gene knockout contributes to inactivation of CD4^+^ T cells to decrease IFN-γ expression and suppress DC development, maturation and secretion of inflammatory cytokines, which leads to inhibition of corneal allograft rejection.

In summary, JAK2 gene knockout is able to inhibit the activation of CD4^+^ T cells to diminish the expression of IFN-γ, which contributes to suppression of the innate immune functions of DCs such as development, maturation and secretion of inflammatory cytokines, thereby reducing corneal allograft rejection (Fig. 5). This finding may provide a theoretical basis for further understanding the mechanism of corneal allograft rejection and provide new ideas and theoretical basis for the development of new and more effective corneal transplantation anti-rejection strategies.

## Materials and methods

### Establishment of corneal allograft model in mice

The experimental mice (purchased from Hunan SJA laboratory animal co., ltd, Changsha, Hunan) were clean mice in specific-pathogen-free grade (donor: C57BL/6; receptor: BALB/c mice; 6–8 weeks old; three corneal allograft model mice and three normal control mice) (the method of model construction was described in the following method) were selected for high-throughput sequencing.

The operation steps were as follows: (1) Anesthesia: 5% sodium pentobarbital was diluted to 0.5% sodium pentobarbital. The body weight of each mouse was measured using an electronic balance before the operation, and the dose used for each mouse was estimated based on the body weight. Next, 0.4 mL/g 0.5% sodium pentobarbital was injected intraperitoneally into mice. (2) Preoperative preparation: 30 min before the operation, the eyes of donor mice and the right eyes of the recipient mice were given 0.5% compound tropicamide eye drops 2–3 times to fully dilate the pupil. The eyelids, eyelashes and periocular fur of the eyes of donor mice and the right eyes of the recipient mice were cut off. Subsequently, 0.1 mL 0.5% tetracaine eye drops were dropped into the eyes of donor mice and the right eyes of the recipient mice for topical anesthesia and the conjunctival sac was washed with normal saline until there was no residual foreign body. During the operation, the eyes were kept up and the respiratory tract unobstructed. The skin around the eyes was disinfected twice with Anil iodine, and 0.1 mL of thalidol eye drop was used to prevent infection. Disinfectant towels were laid routinely for operation. (3) The operation procedures of corneal allograft transplantation: the corneal graft imprint of donor mice were drilled with a trephine in 2.0-mm diameter. The corneal puncture knife was used to puncture into the anterior chamber at the 3 o’clock direction of the cornea at a puncture angle not to damage the lens. After successful puncture, the puncture site was expanded with Venus scissors to a size that a syringe needle could get in. In order to maintain anterior chamber depth, sodium hyaluronate viscoelastic agent was injected into the anterior chamber in time, with prevention of anterior chamber collapse caused by the excessive outflow of aqueous humor. Venus scissors were used to cut the corneal grafts clockwise according to the corneal imprints and the grafts were put in the culture dish with normal saline. After the right eye corneal graft (diameter: 1.5 mm) of recipient mice was made by the same method, the prepared donor graft was gently moved to the center of the graft bed of recipient mice, and 8–10 stitches were intermittently sutured using 11–0 suture. (4) Postoperative nursing: after the operation, the conjunctival sac of the mice was smeared with thalidol eye ointment, and the eyelids were sutured two stitches with 10.0 suture. After 3 days of single cage feeding, the eyelids were opened by removing the suture. After operation, the mice were given thalidol eye drops, 0.1 mL/time, once a day, to prevent infection. The corneal sutures were removed 7 days after the operation. The operation was performed by the same operator. Intraoperative anterior chamber hemorrhage, postoperative iris synechia, and cataract were considered as surgical failure, and those mice were not included in the experimental group; in that case, experimental animals were supplemented in time.

### Transcriptome high-throughput sequencing

Trizol kits (Invitrogen, Carlsbad, CA, USA) were used to extract total RNA from corneal tissue of normal mice and corneal allograft model mice (three biological replicates in each group). The purity, concentration, and integrity of RNA samples were detected in time after digestion of total RNA to ensure the use of high-quality RNA for transcriptome sequencing. Computer testing was performed on the Illumina Next CN500 high-throughput sequencing platform, and the set value of sequencing was read using PEl50. The FASTQ software was used to control the quality of the obtained data, that was, clean data were obtained after the removal of the connectors and low-quality sequences in raw data. The sequences were aligned to the mouse reference genome using the Hisat2 software, and then the gene expression was quantified using R software package to obtain the gene expression matrix.

### Construction of JAK2 knockout mice

Cre^+/+^-JAK2^fl/fl^ mice were selected from the hybrid offspring of Cre-ERT2 transgenic mice and JAK2^fl/fl^ mice. The mice were reared under 12-h light/dark cycles. The male Cre^+/+^-JAK2^f1/f1^ mice that had undergone genotyping were injected subcutaneously with tamoxifen for 5 days at 8-week old. Tamoxifen was freshly prepared with corn oil before injection. Two days after the last injection, mice were used for the experiment. The JAK2^fl/fl^ mice with conditional JAK2 gene knockout were first constructed by Krempler etc. [27]. Through the Cre-lox system, JAK2 gene can be knocked out in adult mice, and the technology can effectively avoid the death caused by the knockout of JAK2 in the embryo stage. In this mouse genome, the upstream and downstream of the first exon of JAK2 were introduced into loxP sequence, the recognition site of Cre recombinase. Under the action of Cre recombinase, the gene fragment between the two loxP sequences will be removed, leading to the failure of JAK2 to express functional protein. Cre-ERT2 transgenic mice were formed by the fusion of Cre recombinase and mutant estrogen receptor protein. It could be activated by tamoxifen but not by estrogen. The fusion protein is driven by the human ubiquitin C promoter. Cre recombinase was activated by tamoxifen injection, which could remove JAK2 from Flox.

### Intervention of JAK2 knockout mice

The study enrolled 18 JAK2 knockout mice (C57BL/6 strain; 6–8 weeks old, specific-pathogen-free grade) and 18 BALB/c mice (6–8 weeks old, specific-pathogen-free grade). The right eyes of the recipient mice were used as the operated eyes, and for the donor mice, both eyes were used. In the wild-type (WT) group, BALB/c mice were used as donors; the C57BL/6 mice were used as the recipients and the right eyes were subjected to corneal allograft transplantation. In JAK2 gene knockout group, BALB/c mice were used as donors; the JAK2 knockout mice were used as the recipients and right eyes were subjected to corneal allograft transplantation.

### Observation of corneal allograft rejection

The transparency of the corneal grafts was observed for consecutive 8 weeks (twice a week) by the same operator under a slit lamp microscope at 72 h after operation. The rejection was scored according to the following criteria: [28] 0 point: the corneal graft was transparent; 1 point: slight epithelial turbid; 2 points: mild matrix turbidity, pupil margin, and iris vessels were visible; 3 points: only some pupil margin was found, with medium matrix turbidity; 4 points: the anterior chamber was only visible, with deep matrix layer turbidity; 5 points: the anterior chamber was not visible and the matrix layer was completely turbid. According to the standard, the degree of rejection was evaluated quantitatively: at 2 weeks and 2 weeks ago, a corneal score ≥3 indicated rejection; after 2 weeks, a corneal scor ≥2 was considered as rejection. Cornea without rejection for consecutive 56 days was considered to permanently survive.

### Hematoxylin and eosin (HE) staining

On the 14th day after the operation, 6 mice in each group were used for histopathological examination. In brief, after intraperitoneal injection of a lethal dose of 0.5% sodium pentobarbital to anesthetize the mice, the mouse eyeballs were immediately collected and fixed in an Eppendorf tube containing paraformaldehyde solution. The collected eyeballs were then dehydrated, cleared, and paraffin-embedded. The wax blocks were sliced into 4-μm sections using a slicer, immersed in warm water, and completely dried in an oven, and immediately dewaxed in xylene, followed by rehydration, staining, dehydration, clearing, and sealing.

### Immunohistochemical staining

On the 14th day after the operation, six mice in each group were used for immunohistochemical staining. In brief, after intraperitoneal injection of a lethal dose of 0.5% sodium pentobarbital to anesthetize the mice, the mouse eyeballs were immediately collected and immersed in an Eppendorf tube containing paraformaldehyde solution for fixation. The collected eyeballs were then dehydrated, cleared, and paraffin-embedded. The wax blocks were cut into slices with a thickness of about 4-μm using a slicer and then placed in warm water. Subsequently, the slices were completely dried in an oven and immediately put into xylene to dissolve and dewax, followed by rehydration, and blockade of endogenous enzymes. After antigen repair, the slices were incubated with primary and secondary antibodies, stained with DAB, counterstained, dehydrated and sealed.

### Reverse transcription-quantitative polymerase chain reaction (RT-qPCR)

Six mice in each group were randomly euthanized 14 days after the operation, and corneas were collected and put into a 1.5 mL centrifuge tube. The total RNA was extracted using Trizol kits (Invitrogen) and then reverse-transcribed into complementary DNA (cDNA) according to the instructions of TaqMan microRNA Assays Reverse Transcription Primer (4427975, Applied Biosystems, Carlsbad, CA, USA). Next, 5 μL of cDNA products were obtained as a template for PCR amplification. β-Actin was used as an internal parameter for mRNAs. The relative difference of gene expression was calculated using 2^−ΔΔCT^ method. The primer sequence is shown in Table S2 (primer design was carried out using the primer design function provided by NCBI).

### Western blot

The total protein of mouse corneas was extracted with radioimmunoprecipitation assay lysate containing phenylmethylsulfonyl fluoride (R0010, Solarbio, Beijing, China). The total protein was incubated on ice for 30 min at 12000 r/minute, followed by centrifugation at 4 °C for 10 min to obtain the supernatant. BCA kits (23225, Pierce, Rockford, IL, USA) were used to determine the protein concentration of each sample, which was further adjusted with deionized water. Next, 50 µg protein samples were separated by 10% sodium dodecyl sulfate-polyacrylamide gel electrophoresis gel (P0012A, Beyotime, Shanghai, China) at 80 V for 2 h. The protein samples were then transferred to a polyvinylidene fluoride membrane (ISEQ00010, Millipore, Billerica, MA, USA) with wet transfer method, which was blocked with Tris-buffered saline with 0.5% Tween 20 buffer containing 5% skimmed milk powder for 2 h. The primary antibody was added to the membrane for overnight incubation at 4 °C. Subsequently, the membrane was incubated with goat anti-rabbit against immunoglobulin G (IgG) antibody labeled with horseradish peroxidase (HRP) (Beijing Zhongshan Biotechnology Co. Ltd., Beijing, China, diluted at 1:5000). Afterwards, the membrane was developed by the enhanced chemiluminescence test kit (BB-3501, Amersham Biosciences, Piscataway, NJ, USA) and exposed in a gel imager. The relative protein expression was expressed by the ratio of the gray value of the corresponding protein band to that of the glyceraldehyde-3-phosphate dehydrogenase protein band.

### Isolation of DCs cells from mouse bone marrow

The mice were euthanized through cervical dislocation, and the femur and tibia were stripped out under aseptic conditions. The bone marrow cells were washed with Hank’s solution and phosphate-buffered saline (PBS), and the red blood cells were lysed with 0.83% Tris-NH_4_Cl, followed by two rinses with 1640 culture solution. The cell concentration was adjusted to 1 × 10^6^ cells/mL, and then the cells were added with recombinant murine granulocyte-macrophage colony-stimulating factor (rmGM-CSF) (1000 U/mL) and RMLL-4 (500 U/mL) in a 24-well culture plate for incubation in an incubator at 37 °C with 5% CO_2_. On the 3rd day, the plate was shaken gently, and most of the suspended cells were absorbed off. The same amount of culture medium was added to the cells, and the cytokines were replenished. Half medium was changed every other day and the suspended or adherent cells were collected on the 7th day.

### Enzyme-linked immunosorbent assay (ELISA)

The culture supernatant of bone marrow-derived DCs (BMDCs) was obtained. The inflammatory factors interleukin (IL)-2, IL-6, IL-10, IL-12 and tumor necrosis factor-α (TNF-α) were determined according to the instructions of ELISA kits. The standard curve was drawn and the content of inflammatory factors was calculated.

### Flow cytometry

On the 14th day after the operation, six mice with different treatments were selected, and the proportion of immune cells in ipsilateral cervical lymph nodes of mice was detected by flow cytometry. After the mice were anesthetized through intraperitoneal injection of 0.5% sodium pentobarbital, the ipsilateral cervical lymph nodes (about four available lymph nodes in each mouse) were collected immediately.

(1) The mice were euthanized through cervical dislocation. After 75% alcohol was sprayed on the surface, the limbs were fixed on the dissecting table with pins. The mouse neck skin was pinched with forceps, the tissue was cut open, and the skin was stripped out. The neck lymph nodes of mice were carefully searched and the lymph nodes were collected through blunt separation. The lymph nodes were placed in Eppendorf tubes containing PBS balanced buffer.

(2) Preparation of lymph node single-cell suspension: the lymph nodes collected in the above steps were transferred to a 200-mesh sieve, which was placed in a clean glass plate. Next, 5 mL PBS was dripped on the lymph nodes, which were gently ground with a 5 mL syringe (In this process, physiological saline was continuously drawn from the plate to wash the sieve to ensure that no cells were left out). The sieve was removed and the cell suspension in the plate was transferred into a 1.5 mL Eppendorf tube, followed by centrifugation at room temperature at 3000 rpm for 3 min.

(3) Detection of cell surface molecules: 1 μL of the above single-cell suspension was taken out and diluted, followed by cell counting. The concentration of cell suspension was recorded, and centrifugation was performed at 4 °C for 5 min to screen the myeloid cells. The cells were resuspended with flow cytometry staining buffer at a concentration of 10^6–7^/mL. Subsequently, 100 μL of the suspension was collected, and incubated with an appropriate amount of surface flow cytometry antibody at room temperature in darkness for 15 min. Finally, resuspension was performed using 150 μL cytometry staining buffer, followed by detection.

### Statistical analysis

All data were processed utilizing SPSS 21.0 statistical software (SPSS, IBM, Armonk, NY, USA). The measurement data from three independent experiments were expressed by mean ± standard deviation. Comparisons between two groups with normal distribution and homogeneous variance were performed using an unpaired *t*-test. Data between multiple groups were compared using one-way analysis of variance (ANOVA), followed by the Tukey post hoc test, and those at different time points were compared by repeated measures of ANOVA. *p* < 0.05 indicated the difference was statistically significant.

## Supplementary information

WB figures

Supplemental Tables

## Data Availability

The data that support the findings of this study are available on request from the corresponding author.
