# Supplementary figures and images for: JAK2 gene knockout inhibits corneal allograft rejection in mice by regulating dendritic cell-induced T cell immune tolerance
Source: Cell Death Discov. 2022 Jun 16;8:289. doi: 10.1038/s41420-022-01067-5 (PMC9203759; doi:10.1038/s41420-022-01067-5)

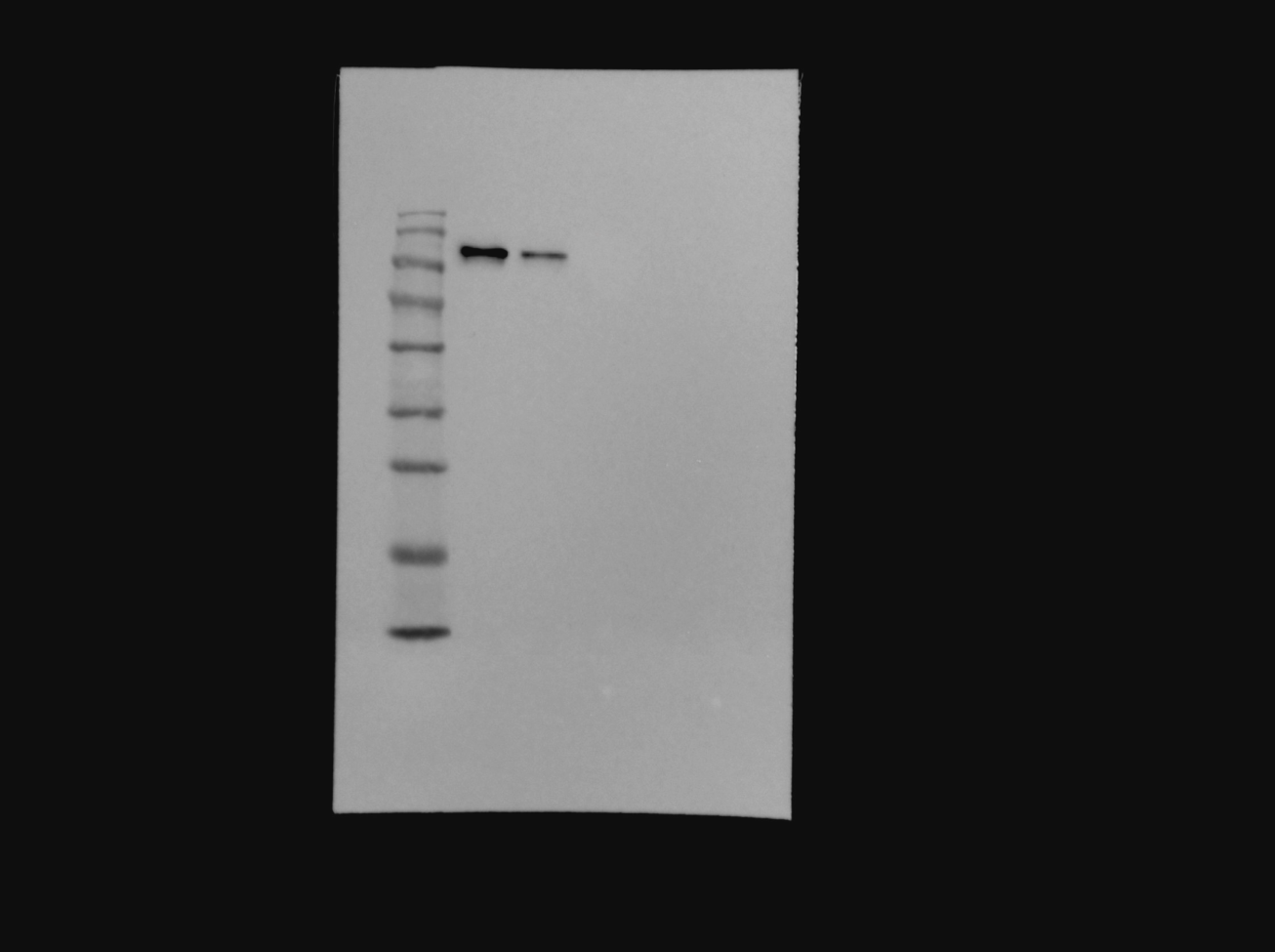
Figure 3B


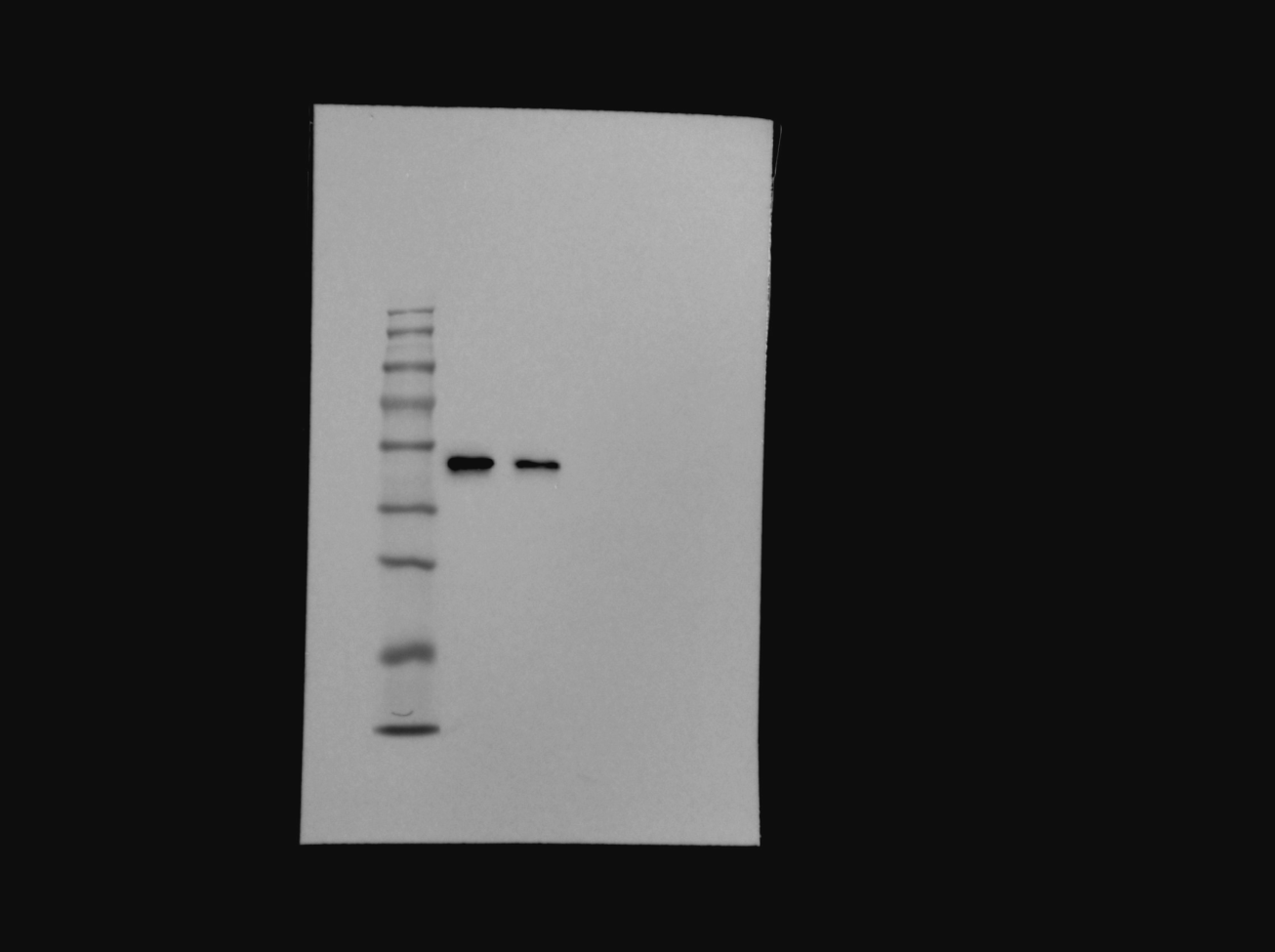
Figure 3G

Supplement: Supplementary file 1 — WB figures [file 41420_2022_1067_MOESM1_ESM.docx]
