# Supplementary material for: JAK2 gene knockout inhibits corneal allograft rejection in mice by regulating dendritic cell-induced T cell immune tolerance
Source: Cell Death Discov. 2022 Jun 16;8:289. doi: 10.1038/s41420-022-01067-5 (PMC9203759; doi:10.1038/s41420-022-01067-5)
Supplement: Supplementary file 2 — Supplemental Tables [file 41420_2022_1067_MOESM2_ESM.docx]

**Table S1 Raw data about the content obtained from the sequencing data**

|  | logFC | logCPM | PValue | FDR |
| --- | --- | --- | --- | --- |
| Gm28884 | 7.665711 | 10.18103 | 1.35E-08 | 5.17E-05 |
| Ncbp3 | 5.908251 | 7.895406 | 2.91E-06 | 0.005569 |
| Dhx8 | -7.59995 | 7.11631 | 5.53E-06 | 0.007053 |
| Wnt9b | -10.0757 | 11.66051 | 9.77E-06 | 0.009356 |
| Hoxb5 | -9.26997 | 8.740363 | 2.53E-05 | 0.019378 |
| Gm42642 | 4.3438 | 6.068259 | 3.38E-05 | 0.02087 |
| Glra1 | 4.866865 | 10.34675 | 4.36E-05 | 0.02087 |
| Jak2 | -8.45903 | 8.623227 | 3.84E-05 | 0.02087 |
| Clpx | -6.0594 | 9.723877 | 5.00E-05 | 0.021292 |
| Dnah5 | 5.03693 | 7.039212 | 5.71E-05 | 0.021456 |
| Rpl31-ps7 | -4.46796 | 5.899342 | 6.16E-05 | 0.021456 |
| Doc2a | 7.980654 | 9.560471 | 9.27E-05 | 0.024282 |
| Myh13 | 7.723312 | 9.985978 | 9.37E-05 | 0.024282 |
| Gm11597 | 7.370085 | 8.917535 | 9.91E-05 | 0.024282 |
| Fbxo40 | -6.34113 | 7.287565 | 9.17E-05 | 0.024282 |
| Prlr | -7.20251 | 8.250395 | 0.000101 | 0.024282 |
| Ino80e | 7.550081 | 9.135766 | 0.000129 | 0.027853 |
| Carmil1 | -5.43334 | 7.674988 | 0.000137 | 0.027853 |
| Stx5a | -5.43668 | 10.45519 | 0.000138 | 0.027853 |
| Igfbp2 | 5.092789 | 8.140458 | 0.000146 | 0.027864 |
| Ctr9 | -5.85747 | 6.230734 | 0.000192 | 0.035072 |
| Ttn | 4.882441 | 11.68954 | 0.000217 | 0.037733 |
| Col1a2 | 5.954205 | 13.01376 | 0.000244 | 0.04061 |
| Atic | 4.622928 | 8.017766 | 0.000275 | 0.043928 |
| Olfr943 | -3.91684 | 5.749182 | 0.000289 | 0.04433 |
| Vmn1r185 | 7.130688 | 8.681789 | 0.000355 | 0.048821 |
| Apoc4 | 6.876989 | 8.433932 | 0.000357 | 0.048821 |
| Lama1 | -4.30942 | 4.306362 | 0.000348 | 0.048821 |
| G3bp1 | 4.617688 | 9.868325 | 0.00041 | 0.051356 |
| Myh8 | 7.57137 | 9.347554 | 0.000431 | 0.051356 |
| Esp16 | 3.936102 | 16.25781 | 0.000451 | 0.051356 |
| Krt73 | 5.419946 | 7.961711 | 0.000456 | 0.051356 |
| Ikbkg | 4.446472 | 8.730141 | 0.000469 | 0.051356 |
| Nemf | -3.63619 | 16.76551 | 0.000454 | 0.051356 |
| Slc37a2 | -4.25726 | 4.27087 | 0.000467 | 0.051356 |
| Itga3 | 5.6953 | 11.36029 | 0.000518 | 0.054115 |
| Tra2a | 3.347278 | 5.440349 | 0.000529 | 0.054115 |
| Ltbp3 | -4.3199 | 14.09347 | 0.000537 | 0.054115 |
| Sorbs3 | -3.92201 | 10.21271 | 0.000572 | 0.056116 |
| Hirip3 | 6.456163 | 8.02497 | 0.000591 | 0.056598 |
| Zfp3 | 5.64992 | 7.445774 | 0.000622 | 0.056727 |
| Gm43811 | -4.24949 | 5.92661 | 0.000618 | 0.056727 |
| Gm38200 | -3.34649 | 5.45011 | 0.00064 | 0.057009 |
| Pdk4 | 3.328219 | 5.424817 | 0.000761 | 0.065917 |
| Ncan | -4.06221 | 4.723463 | 0.000775 | 0.065917 |
| Aftph | 3.032942 | 5.471423 | 0.000816 | 0.067931 |
| R74862 | 6.033988 | 7.737828 | 0.000859 | 0.069994 |
| Ccdc141 | 5.65517 | 7.964331 | 0.000904 | 0.070957 |
| Zfp629 | 4.882021 | 8.675022 | 0.000908 | 0.070957 |
| Gm10309 | 4.270921 | 8.094876 | 0.000954 | 0.073073 |
| Sipa1l3 | 4.881823 | 6.763175 | 0.001039 | 0.07804 |
| Gm45889 | 5.910819 | 7.501024 | 0.001068 | 0.07864 |
| Irf7 | 8.014935 | 9.672371 | 0.001089 | 0.078687 |
| Gm3924 | 3.308973 | 5.378213 | 0.001208 | 0.082631 |
| Slc35a3 | -3.18958 | 6.676931 | 0.001187 | 0.082631 |
| Gm49866 | -3.19073 | 4.988829 | 0.001195 | 0.082631 |
| Gm15593 | 3.327166 | 7.583278 | 0.001278 | 0.085853 |
| Muc16 | 4.682044 | 6.546661 | 0.001338 | 0.088112 |
| Bora | 4.425396 | 7.805428 | 0.001358 | 0.088112 |
| Hoxd9 | -3.09824 | 5.099542 | 0.00139 | 0.088704 |
| Etv1 | 3.843025 | 6.89423 | 0.001439 | 0.09034 |
| Ces2a | 3.392251 | 7.846079 | 0.001501 | 0.091877 |
| Gvin1 | 3.139031 | 6.482425 | 0.001512 | 0.091877 |
| Aqp3 | 4.035807 | 5.798549 | 0.001549 | 0.092667 |
| Gm48718 | -3.20656 | 4.706345 | 0.001591 | 0.093728 |
| Gm5921 | -3.80944 | 5.302981 | 0.001646 | 0.095509 |
| Gm50317 | -3.287 | 6.333831 | 0.001681 | 0.09607 |
| Slc4a8 | 3.755513 | 5.924724 | 0.001795 | 0.100286 |
| Gm14881 | -3.7713 | 4.438281 | 0.001807 | 0.100286 |
| Mrpl33 | -3.49121 | 5.992615 | 0.001872 | 0.102378 |
| Vim | 3.759862 | 8.657712 | 0.001933 | 0.10282 |
| Mnat1 | -2.99961 | 5.273408 | 0.001922 | 0.10282 |
| Slc17a7 | 3.302023 | 6.256273 | 0.002019 | 0.102983 |
| Ankrd11 | 3.19267 | 6.925706 | 0.002044 | 0.102983 |
| Cntrob | -4.55876 | 4.478023 | 0.00202 | 0.102983 |
| Txndc9 | -3.03054 | 4.755744 | 0.002029 | 0.102983 |
| Eapp | -3.37274 | 9.639188 | 0.002071 | 0.102983 |
| 2900060N12Rik | -3.81063 | 5.662457 | 0.002165 | 0.106276 |
| Tmem45a | 3.714851 | 5.568168 | 0.002194 | 0.10633 |
| Rai14 | 3.485344 | 5.783342 | 0.002254 | 0.106904 |
| Stac | -4.09545 | 4.162918 | 0.002261 | 0.106904 |
| Gm26244 | -3.14336 | 6.508952 | 0.002302 | 0.107469 |
| Gm8702 | -3.34478 | 6.776442 | 0.002548 | 0.117566 |
| Ptov1 | 5.469249 | 7.082531 | 0.0028 | 0.127322 |
| Gm29585 | -2.95335 | 4.855392 | 0.002826 | 0.127322 |
| Svip | -3.96204 | 4.077664 | 0.002889 | 0.128627 |
| Mmp13 | 5.983451 | 7.569139 | 0.002963 | 0.128904 |
| Sh3rf2 | -3.00636 | 6.003673 | 0.002935 | 0.128904 |
| Neb | 3.996639 | 8.469825 | 0.003024 | 0.129147 |
| Arl5a | 5.289923 | 6.915143 | 0.00308 | 0.129147 |
| Gm4316 | 5.101782 | 8.779599 | 0.003119 | 0.129147 |
| Cav1 | 3.194611 | 7.247968 | 0.003137 | 0.129147 |
| Fmc1 | -3.02046 | 4.661979 | 0.003102 | 0.129147 |
| Fn1 | 3.482212 | 7.328347 | 0.003265 | 0.130216 |
| Sparc | 3.758745 | 6.643361 | 0.003277 | 0.130216 |
| Chaf1b | -4.68628 | 7.048151 | 0.003213 | 0.130216 |
| Grin2b | -4.45107 | 5.329676 | 0.003299 | 0.130216 |
| Picalm | -3.07276 | 5.041046 | 0.003468 | 0.135497 |
| Rps6ka5 | -2.8607 | 4.600429 | 0.003536 | 0.136749 |
| Sec14l2 | 5.49459 | 7.784215 | 0.003635 | 0.137805 |
| Ccdc125 | -3.13841 | 5.28822 | 0.003634 | 0.137805 |
| Uap1 | 3.005624 | 5.174914 | 0.00385 | 0.144034 |
| Cyp2f2 | 6.208069 | 7.783739 | 0.003889 | 0.144034 |
| Olfr263 | 6.180528 | 7.757288 | 0.003922 | 0.144034 |
| Mmp20 | 6.165704 | 7.743054 | 0.00395 | 0.144034 |
| Sncg | 4.567864 | 6.258077 | 0.00407 | 0.145863 |
| Ntm | -3.99687 | 5.314943 | 0.004076 | 0.145863 |
| Gm20628 | 2.574945 | 4.835374 | 0.004164 | 0.14764 |
| Art4 | 2.572453 | 5.557287 | 0.004282 | 0.150436 |
| Arhgap42 | 2.693655 | 6.20457 | 0.004568 | 0.154577 |
| Junos | -2.88007 | 5.15276 | 0.004505 | 0.154577 |
| Galnt18 | -2.70677 | 5.844085 | 0.004524 | 0.154577 |
| Rps7 | -4.28295 | 8.586671 | 0.004579 | 0.154577 |
| Gm42523 | -4.03055 | 4.119798 | 0.004602 | 0.154577 |
| BC043934 | -3.47391 | 5.564428 | 0.004732 | 0.157561 |
| Tpm2 | 3.290844 | 10.11958 | 0.005027 | 0.164217 |
| Igfn1 | 3.659122 | 6.236802 | 0.005168 | 0.164217 |
| Car8 | 3.608556 | 6.90857 | 0.005232 | 0.164217 |
| Mlc1 | -2.97575 | 6.621622 | 0.005109 | 0.164217 |
| F930017D23Rik | -3.89463 | 7.795464 | 0.005178 | 0.164217 |
| Tmem64 | -3.38602 | 7.304106 | 0.005199 | 0.164217 |
| Mybpc3 | -2.47385 | 4.891191 | 0.005204 | 0.164217 |
| Gm44752 | 2.63163 | 5.014917 | 0.005444 | 0.169479 |
| Cenpo | 2.516702 | 4.795092 | 0.005612 | 0.172377 |
| Gm12722 | 2.741478 | 5.249099 | 0.005732 | 0.172377 |
| Lmod2 | 4.641634 | 6.322949 | 0.005798 | 0.172377 |
| Tmem44 | -2.44814 | 5.708744 | 0.005696 | 0.172377 |
| Gm9967 | -2.43264 | 5.014536 | 0.005791 | 0.172377 |
| Gm47338 | -3.8659 | 4.866058 | 0.005822 | 0.172377 |
| Gm49883 | -2.69825 | 5.957594 | 0.005852 | 0.172377 |
| Rian | -4.05272 | 5.359962 | 0.005954 | 0.174042 |
| Ap1m2 | 2.692586 | 5.981097 | 0.00603 | 0.174909 |
| Srpx | -3.6785 | 5.629123 | 0.006274 | 0.180633 |
| Jup | 4.007962 | 5.772062 | 0.006499 | 0.183041 |
| Capsl | 3.630178 | 5.461142 | 0.006501 | 0.183041 |
| Gm44684 | -3.77491 | 3.962033 | 0.00644 | 0.183041 |
| Gm21814 | -2.76753 | 6.026055 | 0.006556 | 0.183244 |
| Tent2 | -3.82716 | 3.993422 | 0.006745 | 0.187157 |
| Gm15759 | 5.227239 | 6.85636 | 0.006821 | 0.18791 |
| Gm12315 | 5.178658 | 6.811418 | 0.006969 | 0.189258 |
| Cacng7 | -3.59741 | 4.318632 | 0.00692 | 0.189258 |
| 1700025G04Rik | -2.63161 | 5.553892 | 0.007073 | 0.190719 |
| Gm42885 | -2.79444 | 5.747697 | 0.00727 | 0.194667 |
| Men1 | 3.95502 | 7.858439 | 0.007422 | 0.19661 |
| Tyk2 | -3.77389 | 5.598541 | 0.007494 | 0.19661 |
| Glis2 | -2.44057 | 4.698788 | 0.007497 | 0.19661 |
| Vmn2r117 | 2.538799 | 7.852958 | 0.007734 | 0.199126 |
| Alg11 | 3.024369 | 5.5149 | 0.007749 | 0.199126 |
| Celsr2 | -2.73347 | 5.151277 | 0.007654 | 0.199126 |
| Dcun1d3 | 3.560925 | 5.404701 | 0.008047 | 0.204047 |
| Ecpas | -2.97272 | 12.3515 | 0.008016 | 0.204047 |
| Map4k2 | 3.548755 | 7.395521 | 0.00816 | 0.204594 |
| Coro2a | 2.499554 | 5.662014 | 0.008175 | 0.204594 |
| Gm26743 | 4.241866 | 5.970907 | 0.008552 | 0.209795 |
| Trim24 | 2.458602 | 5.751815 | 0.008643 | 0.209795 |
| C1qtnf7 | -2.92028 | 6.746153 | 0.008536 | 0.209795 |
| Gm15947 | -2.32227 | 5.511924 | 0.008558 | 0.209795 |
| Edar | -2.41502 | 7.00689 | 0.008657 | 0.209795 |
| Ankrd28 | 2.677851 | 4.935143 | 0.008778 | 0.211384 |
| Gm20461 | 3.623986 | 5.492164 | 0.009099 | 0.216962 |
| Chd7 | 2.497096 | 7.989878 | 0.009123 | 0.216962 |
| Acot10 | -3.01336 | 5.253932 | 0.009405 | 0.2223 |
| Tmem30a | 3.652636 | 6.243071 | 0.009867 | 0.223714 |
| Ppp1r3a | 3.707994 | 5.522148 | 0.009867 | 0.223714 |
| Sh3glb2 | 2.298527 | 5.024646 | 0.009874 | 0.223714 |
| Nr1h3 | -2.42308 | 6.519482 | 0.009533 | 0.223714 |
| Spz1 | -2.60766 | 5.539554 | 0.009681 | 0.223714 |
| Clic3 | -2.84822 | 5.754044 | 0.009778 | 0.223714 |
| Gm49702 | -3.23153 | 6.354549 | 0.009864 | 0.223714 |
| Osgin2 | -2.57978 | 4.700075 | 0.010173 | 0.227523 |
| Ell2 | -3.14252 | 5.819307 | 0.010196 | 0.227523 |
| Rrm2b | -2.50243 | 5.180384 | 0.010508 | 0.230596 |
| Gm48302 | -3.12383 | 5.422341 | 0.011148 | 0.237145 |
| 4933405O20Rik | -3.31955 | 4.140363 | 0.011506 | 0.24075 |
| Atf2 | -3.48059 | 5.251576 | 0.011602 | 0.241439 |
| Gm38067 | -2.70097 | 4.972259 | 0.012008 | 0.244569 |
| Zfp64 | -2.29668 | 5.138316 | 0.012423 | 0.250805 |

**Table S2. Primer sequences for RT-qPCR**

| Gene | Primer sequences (5’-3’) |
| --- | --- |
| JAK2 | F: TACGCACCTGAATCCTTGAC |
|  | R: GTATGTGAAAAGTTCGTATA |
| IFN-γ | F: ACATGAAAATCCTGCAGAGCC |
|  | R: TGACGCTTATGTTGTTGCTG |
| β-actin | F: TGTTACCAACTGGGACGACA |
|  | R: CTTTTCACGGTTGGCCTTAG |

Note: JAK2, Janus kinase 2 gene; IFN-γ, interferon γ; RT-qPCR, reverse transcription-quantitative polymerase chain reaction.
